# Preferences for mHealth Features to Support Engagement in the HIV Preexposure Prophylaxis Cascade Among Men Who Have Sex With Men in Peru: Cross-Sectional Online Survey

**DOI:** 10.2196/84982

**Published:** 2026-07-14

**Authors:** Jorge A Gallardo-Cartagena, Dora Leidy Geraldine German-Quiñones, Kelika Anne Konda, Susan Buchbinder, Jorge Sanchez, Frederick L Altice

**Affiliations:** 1 Centro de Investigaciones Tecnológicas, Biomédicas y Medioambientales, Universidad Nacional Mayor de San Marcos, José Santos Chocano 199, Bellavista, Callao, 07006, Peru, +51 950306764; 2 Keck School of Medicine, University of Southern California, Los Angeles, CA, United States; 3 San Francisco Department of Public Health, San Francisco, CA, United States; 4 Center for Interdisciplinary Research on AIDS, Yale School of Medicine, Yale University, New Haven, CT, United States

**Keywords:** HIV prevention, preexposure prophylaxis, mHealth, digital health, smartphones, human-centered design, implementation science, men who have sex with men, Peru, Latin America, mobile health

## Abstract

**Background:**

Despite policy-level progress, implementation of oral HIV preexposure prophylaxis (PrEP) remains limited in Latin America. In Peru, men who have sex with men (MSM) account for most new HIV diagnoses, yet uptake remains low. Widespread smartphone ownership and the use of digital platforms present an opportunity to expand access through mobile health (mHealth) interventions. However, limited data exist on user preferences to guide the design of mHealth tools in Spanish-speaking Latin American settings.

**Objective:**

This study aimed to assess preferences for mHealth features to support PrEP engagement among Peruvian MSM and their association with PrEP cascade stages.

**Methods:**

We conducted a cross-sectional online survey (June-August 2023) among 600 HIV-negative MSM residing in Peru (median age 29, IQR 24-35 years), recruited via Facebook, Instagram, WhatsApp (Meta Platforms, Inc), and Grindr (Grindr LLC). The survey assessed communication platform use, interest in mHealth features measured on a 4-point Likert scale, and PrEP cascade stages. Exploratory factor analysis (principal axis factoring with Promax rotation) identified domains of mHealth preferences, from which median domain scores were calculated. Bivariate analyses used chi-square tests and Wilcoxon rank sum tests. Multivariable logistic regression models (*α*=.05), with covariates selected using stepwise procedures from candidate sociodemographic and behavioral variables, estimated associations between each domain score and PrEP cascade stages, each modeled as a separate binary outcome.

**Results:**

Nearly all participants (589/600, 98.2%) reported owning a smartphone. WhatsApp was the most frequently used and preferred platform for PrEP support, with 547 (91.2%) reporting frequent use and 302 (50.3%) ranking it first. Exploratory factor analysis identified three mHealth preference domains: informational support (Cronbach α=0.94), self-management tools (Cronbach α=0.94), and interactive communication (Cronbach α=0.91). Among participants, 483 (80.5%) had decided to use PrEP, 190 (31.7%) had sought PrEP, and 109 (18.2%) had initiated PrEP. Higher informational support was associated with the decision to use PrEP (adjusted odds ratio [aOR] 4.54, 95% CI 3.36‐6.28; *P*<.001), seeking PrEP (aOR 1.43, 95% CI 1.10‐1.89; *P*=.001), and PrEP initiation (aOR 1.64, 95% CI 1.16‐2.44; *P*=.009). Self-management tools showed similar associations with the decision to use PrEP (aOR 3.23, 95% CI 2.51‐4.22; *P*<.001), seeking PrEP (aOR 1.34, 95% CI 1.06‐1.70; *P*=.02), and PrEP initiation (aOR 1.49, 95% CI 1.11‐2.05; *P*=.01). Interactive communication was associated with the decision to use PrEP (aOR 2.74, 95% CI 2.15‐3.53; *P*<.001) but not with initiation.

**Conclusions:**

Preferences for mHealth features were associated with engagement at multiple stages of the PrEP cascade among MSM in Peru. Informational support features demonstrated the most consistent associations with cascade engagement. These findings provide empirical evidence on user-prioritized digital functions that could support early engagement in HIV prevention services in a Latin American implementation context. Integrating culturally tailored mHealth tools within widely used platforms such as WhatsApp may strengthen early PrEP cascade engagement and support scalable digital strategies for HIV prevention in Peru and similar settings.

## Introduction

Despite important gains in HIV prevention, HIV remains a pressing public health concern across Latin America, with a disproportionately high incidence among gay, bisexual, and other men who have sex with men (MSM), as well as transgender women [1]. In Peru, these populations account for the vast majority of new HIV diagnoses, reflecting persistent gaps in prevention services and structural inequities [2].

Oral preexposure prophylaxis (PrEP) is an evidence-based intervention to prevent HIV acquisition [3]. Although many Latin American countries have begun integrating PrEP into their public health policies, uptake remains limited and uneven [4]. In Peru, fewer than 8000 individuals were using PrEP among an estimated 140,000 who could benefit by 2025 [5]. Gaps along the PrEP cascade, including seeking, initiation, and adherence, underscore the need for scalable, user-centered approaches to support delivery.

Digital communication technologies are widely used among MSM in Latin America [6]. Social media, mobile apps, and geosocial networking platforms are common sources of both social connection and health information [7-9]. This widespread engagement presents opportunities to integrate digital solutions, such as mobile health (mHealth) apps, messaging platforms, social media–based tools, and AI-enabled chatbots, into HIV prevention efforts.

Globally, mHealth interventions have shown promise in promoting HIV testing, linkage to care, and engagement with HIV prevention services, including PrEP uptake and adherence [10-12]. Randomized trials of mHealth apps in the United States have demonstrated measurable effects on HIV testing and progression along the PrEP cascade. For example, HealthMindr-PrEP (Emory University) [13] increased PrEP uptake overall, particularly among individuals with baseline PrEP indications. In a national randomized trial among young sexual minority men, MyChoices (Keymind) [14] increased HIV testing compared with standard prevention services, whereas LYNX (APT Mobility) [14] showed a nonsignificant increase in testing; neither intervention significantly increased PrEP initiation. Other trials have focused on adherence and retention among individuals using PrEP. SMS text messaging–based interventions such as PrEPmate (WelTel Inc) [15] and iTAB (University of California San Diego) [16] improved visit retention and some adherence outcomes, while app-based tools such as the Amsterdam PrEP app (Amsterdam PrEP Project/H-TEAM) [17], PrEP-iT! (San Diego State University) [18], P3 (Florida State University) [19], and DOT Diary (AiCure LLC) [20] demonstrated signals of improved adherence. Messaging platforms have also been leveraged for adherence support, including reminder systems delivered through WeChat (Tencent Mobile International Limited) [21] and weekly text messaging interventions implemented in Brazil [22].

Additional approaches target service navigation and demand generation. Clinic-integrated platforms such as JomPrEP (JomPrEP study team) [23,24] enable HIV self-testing, teleconsultation, and rapid PrEP initiation through mobile interfaces, while digital peer-navigation tools such as PrEPresent (Florida State University) [25] support engagement with PrEP services. Decision-support tools such as MyPrEP (University of Washington) [26] have also been developed to help individuals assess HIV risk and evaluate whether PrEP is appropriate before clinical consultation, and have been associated with improved early PrEP persistence in a randomized trial. AI-enabled chatbot–based conversational agents are also emerging as digital prevention tools. For example, Amanda Selfie (PrEP1519 study team) [27], MYHIV365 (Yale University) [28,29], and CHIA (Brown University) [30] deliver automated HIV prevention information and counseling through interactive chat interfaces and have demonstrated usability and acceptability in early testing phases. Formative work further highlights the importance of culturally tailored design; SaludFindr (Emory University) [31] was developed to address barriers to PrEP uptake among Latino sexual minority men in the United States, and DOT Diary and PrEPmate have been adapted to support adherence among linguistically diverse populations [32]. Ongoing trials such as Combine [33] are also evaluating mobile interventions designed to increase HIV testing and PrEP initiation among underserved populations, including rural MSM in the southern United States.

While this body of work reflects substantial innovation, most evaluated interventions have been implemented in high-income settings, and evidence specific to Latin American health care systems remains limited, particularly for Spanish-speaking populations. Although Spanish is widely spoken in Latin America, communication norms, health system navigation pathways, and colloquial vocabulary vary across countries, potentially limiting the direct transferability of digital tools developed elsewhere [34]. Because these contextual factors may influence how digital interventions are understood and used, assessing user preferences is an important first step before developing or adapting existing mHealth tools for Peru and similar Latin American settings.

To address this gap, we conducted a survey among MSM in Peru to characterize communication technology use and assess preferences for mHealth features to support PrEP engagement. We examined whether interest in distinct domains of mHealth support was associated with engagement in three PrEP cascade stages: (1) decision to start PrEP; (2) actively seeking PrEP services; and (3) PrEP initiation. We hypothesized that greater interest in specific mHealth domains would be associated with a higher likelihood of having reached these stages. Because these stages represent distinct behavioral transitions, digital supports may operate differently at each stage. While grounded in the Peruvian context, these findings may inform the design of culturally responsive, user-centered interventions to improve PrEP engagement across Latin America, with appropriate adaptations for local settings.

## Methods

### Study Design

We conducted a cross-sectional online survey between June 16 and August 31, 2023, to explore the use of communication technologies and preferences for mHealth features to support HIV prevention among Peruvian MSM. The study followed the Journal Article Reporting Standards for quantitative research [35].

### Participant Selection

Respondents were recruited using convenience, nonprobability sampling through paid advertisements on Instagram, Facebook, Grindr, and via unpaid standard posts (“organic content”) shared on social media and WhatsApp groups managed by participating HIV and AIDS service organizations. Recruitment materials targeted MSM aged 18 years and older residing in Peru.

Participants accessed the survey through a secure link and completed it using their own internet-connected electronic devices; no data subsidies were provided. The survey platform required an active internet connection; offline completion was not supported. Electronic informed consent was required before initiating the survey. The survey instrument was administered in Spanish using standard Peruvian terminology reviewed for clarity and cultural appropriateness. Responses were anonymous, and participants were allowed to skip any question they did not wish to answer.

To ensure data integrity, responses were restricted to one submission per IP address, and IP addresses, timestamps, and completion times were reviewed to identify duplicate, automated, or implausibly rapid entries; none were identified.

### Measures and Covariates

#### Survey Design

The 34-item survey included multiple-choice and Likert-scale questions covering sociodemographic and behavioral characteristics, achieved stages in the HIV prevention cascade (refer to Variable Definitions for operational definitions) [36,37], smartphone and internet access, frequency of use of communication channels (eg, phone calls, SMS text messaging, WhatsApp, social media, and internet browsing), and preferences for features of mHealth tools designed to support PrEP use.

#### mHealth Feature Preferences

To assess preferences for mHealth tools to support PrEP use, participants were presented with a series of 11 potential features and asked to rate their level of interest in each. The stem question read, “How interested would you be in receiving the following types of PrEP-related support through your mobile phone?” Responses were collected on a 4-point Likert scale, ranging from “Not interested” to “Very interested.” The list of features was developed based on existing literature and used human-centered design by refining items through input from community partners to ensure local relevance and clarity [15,20,26,32].

The features covered both informational and behavioral support functions. Informational items included general PrEP education (eg, what PrEP is, how it works, and how to take it), more detailed information (eg, whether someone is a good candidate, side effects, and steps to start) [26], and logistical details (eg, where PrEP is offered, clinic hours, frequency of visits, and cost). Behavioral support features included reminders for PrEP appointments and pill-taking, interactive communication through a virtual assistant or a real person [15], and several app-based tools: push notifications to take PrEP, a daily pill tracker, a sexual activity log, and a feature that assesses whether the user is currently protected from HIV based on their reported pill use and sexual behavior [20].

#### Exploratory Factor Analysis

To identify latent constructs representing participant preferences for mHealth features, we conducted an exploratory factor analysis (EFA) on 11 items assessing interest in different functionalities of hypothetical mHealth PrEP support tools [38,39]. Before the EFA, data suitability was evaluated using the Kaiser-Meyer-Olkin measure of sampling adequacy and Bartlett test of sphericity. We used principal axis factoring with Promax (oblique) rotation, given the expected correlation among features. The number of factors retained was based on eigenvalues >1.0, scree plot inspection, parallel analysis, and conceptual clarity. Resulting factors were used to generate composite scores that represented core domains of mHealth tool interest, which were then used in regression analyses.

#### Variable Definitions

##### Preferred Communication Channel Category

The preferred communication channel category for PrEP support was defined according to the participant’s most preferred platform for receiving PrEP-related communication. “Traditional channels” referred to phone calls or SMS text messaging, which do not require smartphone-specific apps. “Digital channels” included WhatsApp, a customized PrEP app, or other messaging or social media platforms such as Telegram (Telegram FZ-LLC), Messenger and Instagram (Meta Platforms, Inc), or TikTok (ByteDance Ltd), all of which require a smartphone and an internet connection.

##### PrEP Cascade Outcomes

PrEP cascade outcomes reflected 3 distinct stages of engagement in the HIV prevention cascade [36,37], selected for their proximity to PrEP uptake and their potential to be directly influenced by mHealth interventions. The decision to use PrEP was defined as responding “Yes” or “Probably yes” to the question “Have you decided to start using PrEP?” Sought PrEP referred to self-reported attempts to obtain PrEP from a clinic or provider. PrEP initiation was defined as having ever started PrEP, including both current and past use.

##### Sociodemographic and Behavioral Covariates

Sociodemographic covariates included age (<25 years vs ≥25 years; based on regional PrEP implementation evidence showing lower engagement among younger individuals [40]), educational level (elementary and secondary vs post-secondary), monthly income (<1000 Peruvian soles [PEN; 1000 PEN=US $268 as of August 15, 2023], equivalent to the minimum monthly wage at the time of the study [41], vs ≥1000 PEN), and city of residence (Metropolitan Lima vs other cities). Behavioral variables referred to the past 6 months and included self-perceived HIV risk (yes or no), engagement in anal sex (yes or no), number of anal sex partners (≤5 vs>5), condom use (always vs not always), transactional sex (yes or no), and recent bacterial sexually transmitted infection (STI) diagnosis (chlamydia, gonorrhea, or syphilis; yes or no).

### Statistical Analysis

#### Analytic Sample Determination

The analytic sample included participants who completed all exposure and outcome measures. Respondents who reported being assigned female at birth, identified as a gender other than cisgender male, or self-reported living with HIV were excluded from the analytic sample. No missing data were observed among the 600 participants included in the analysis; therefore, complete-case analysis was performed.

#### Descriptive Statistics

We summarized participant characteristics using frequencies and percentages for categorical variables and medians with IQRs for continuous variables given nonnormal distributions. Group comparisons used 2-tailed chi-square tests or Fisher exact tests when expected cell counts were <5. Continuous variables were compared using Wilcoxon rank sum tests. To compare preferences across digital communication platforms, we used pairwise 2-tailed Wilcoxon signed-rank tests on participant rankings, applying Bonferroni correction for multiple comparisons. Statistical significance was defined as *α*=0.05.

#### Logistic Regression Analyses

We conducted bivariate and multivariable logistic regression analyses with a logit link to examine associations between interest in mHealth tool features and engagement at 3 distinct stages of the HIV prevention cascade. Interest in mHealth features was operationalized using median composite scores derived from EFA. Each PrEP cascade outcome was modeled independently to reflect conceptually distinct dimensions of PrEP engagement with potentially different determinants. Because the cascade stages were assessed cross-sectionally and were not strictly sequential in all participants, we modeled each stage as a separate binary outcome rather than imposing hierarchical or conditional restrictions that would reduce sample size and could introduce selection bias. This approach allowed us to estimate associations with having reached each cascade stage without assuming temporal ordering or monotonic progression across stages.

For each outcome, stepwise selection based on the lowest Akaike Information Criterion was first applied to sociodemographic and behavioral variables to identify relevant covariates independently for each PrEP cascade stage. Akaike Information Criterion was used to balance model fit and parsimony while reducing the risk of overfitting. Variance inflation factors were examined to assess multicollinearity, with all retained covariates having variance inflation factor <2. The selected variables were then used to adjust separate multivariable logistic regression models for each mHealth composite factor score. Results are presented as odds ratios and adjusted odds ratios (aORs), with 95% CI. As a sensitivity analysis, multinomial logistic regression models were fitted, treating each cascade stage as a categorical outcome to assess whether associations observed in stage-specific models were consistent when modeled jointly. All analyses were conducted using R version 4.3.1 (R Foundation for Statistical Computing).

### Ethical Considerations

The informed consent form and survey instrument (Multimedia Appendix 1) were reviewed and approved in Spanish by the Comité Institucional de Bioética de Vía Libre (approval 9008‐2023 a). Electronic informed consent was obtained before survey access, and a waiver of documentation of written informed consent was issued to preserve participant anonymity. Participants indicated consent by selecting “I agree” before accessing survey items. The consent form clearly stated that the survey was intended for adult MSM living in Peru, and self-identification was confirmed upon accepting the consent. No personally identifying information was collected, and IP addresses were used solely for duplicate detection. All data were stored on secure, password-protected servers accessible only to the research team. Participation was voluntary, and respondents could skip any question or exit the survey at any time without penalty. No financial compensation was provided. Upon completion, participants received information on HIV prevention services and resources. No identifiable images or personal data are included in this paper.

## Results

### Participant Flow

**Figure 1.**
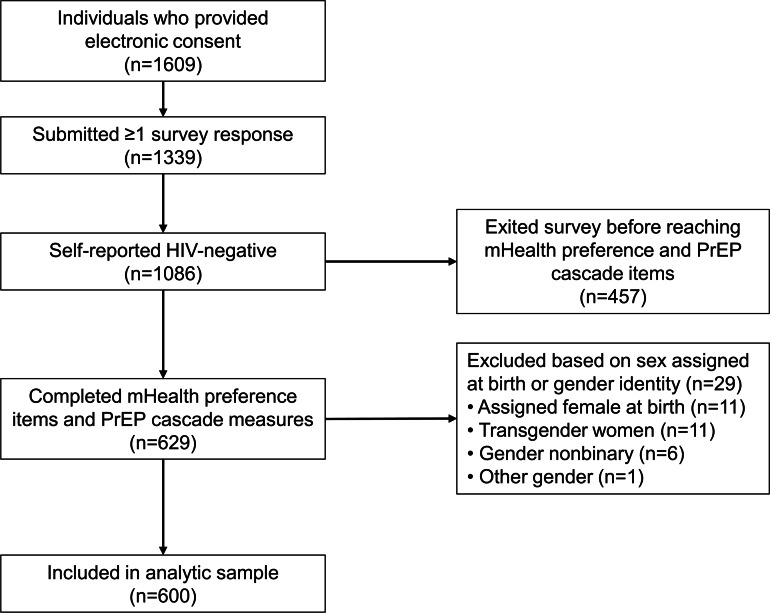
Participant flow diagram for a cross-sectional online survey assessing communication technology use and preferences for mobile health (mHealth) features to support HIV preexposure prophylaxis (PrEP) engagement among HIV-negative men who have sex with men in Peru (June 16-August 31, 2023).

Of the 1609 individuals who provided informed consent, 1339 submitted at least one survey response. Among these, 1086 self-reported being HIV-negative and were retained for further screening. A total of 457 respondents were excluded because they did not complete the required exposure or outcome items due to survey dropout before reaching those sections. Compared to those with incomplete surveys, respondents who completed the full survey were older (median 29.0, IQR 24.0‐35.0 years vs median 27, IQR 21.0‐33.0 years; *P*<.001), more likely to reside in Metropolitan Lima (473/629, 75.2% vs 262/457, 57.3%; *P*<.001), have postsecondary education (494/629, 78.5% vs 328/457, 71.8%; *P*=.01), report higher perceived HIV risk (455/629, 72.3% vs 263/457, 60.9%; *P*=.001), and report more than 5 anal sex partners in the past 6 months (177/629, 35.3% vs 69/457, 25%; *P*=.005). There were no significant differences in sex, gender identity, income, or behavioral characteristics. We further excluded 29 respondents who did not identify as cisgender men, resulting in a final analytic sample of 600 HIV-negative MSM who completed the survey. Participant flow is summarized in Figure 1.

### Participant Characteristics

Participants were primarily recruited through organic content on social media and WhatsApp groups (305/600, 50.8%), followed by paid advertisements on Instagram or Facebook (216/600, 36%) and on Grindr (79/600, 13.2%) (Table S1 in Multimedia Appendix 2). The median age in the final analytic sample was 29 (IQR 24‐35) years, with 152 (25.3%) aged <25 years. Most participants had completed postsecondary education (479/600, 79.8%), and 403 (67.2%) reported a monthly income exceeding 1000 PEN. A total of 450 (75%) resided in Metropolitan Lima. In the past 6 months, 476 (79.3%) engaged in anal sex, 354 (59%) reported condomless sex, and 168 (28%) reported a recent bacterial STI diagnosis. Additionally, 95 (15.8%) engaged in transactional sex, and 440 (73.3%) perceived themselves to be at risk for HIV.

Engagement along the PrEP cascade varied. While 518 (86.3%) participants expressed interest in using PrEP and 483 (80.5%) had made the decision to use it, fewer had taken steps to access it. A total of 190 (31.7%) had sought PrEP, 109 (18.2%) had initiated it, and only 43 (7.2%) were currently using PrEP with self-reported good adherence.

### Communication Behaviors

Smartphone ownership was nearly universal (589/600, 98.2%). Participants reported frequent use of multiple digital platforms, with WhatsApp emerging as the most widely used platform; 547 (91.2%) participants reported using it multiple times per day or throughout the day. Frequent use of internet browsers (498/600, 83%) and social media (405/600, 67.5%) was also common. Phone calls remained widely used, with 432 (72%) participants reporting several calls per day or all-day use. In contrast, SMS text messaging was less popular, with only 193 (32.2%) participants reporting similarly frequent use (Figure S1 in Multimedia Appendix 2).

### Preferred Channels for PrEP Support

**Figure 2.**
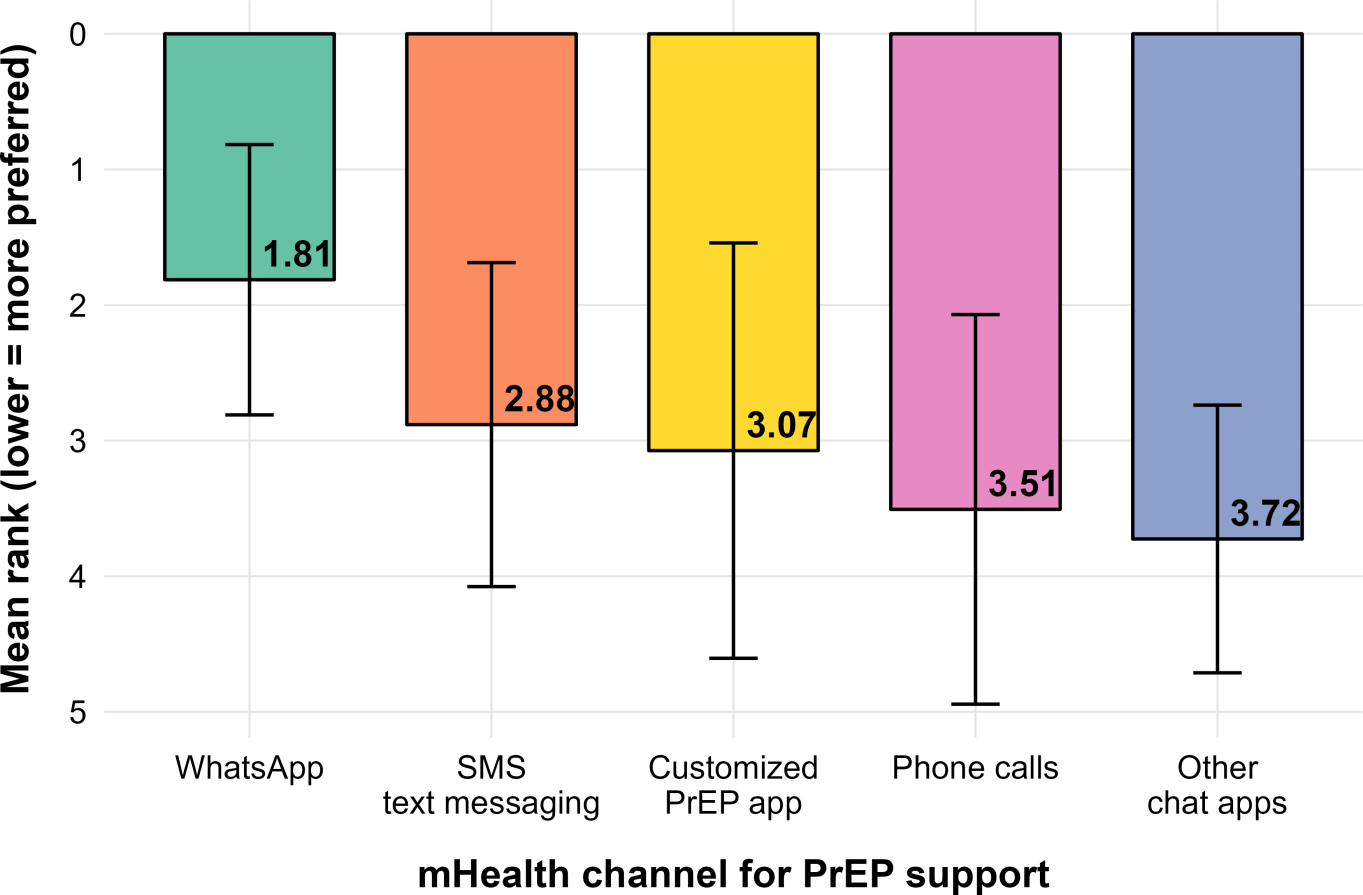
Mean rank scores of preferred communication channels for receiving HIV preexposure prophylaxis–related support among HIV-negative men who have sex with men in Peru (cross-sectional online survey, June 16-August 31, 2023; N=600). Lower mean rank scores indicate greater preference for each communication channel. mHealth: mobile health; PrEP: preexposure prophylaxis.

Participants expressed a strong preference for receiving PrEP-related support through digital channels. As shown in Figure 2, WhatsApp was the most preferred platform, with 302 (50.3%) participants ranking it as their top choice (Figure S2 in Multimedia Appendix 2). This preference was statistically stronger than for any other channel (all adjusted *P*<.001). Preferences across the remaining platforms also differed significantly in nearly all pairwise comparisons. The only exceptions were the comparisons between the customized PrEP app and SMS text messaging (adjusted *P*=.24), and between other chat apps and phone calls (adjusted *P*=.07). When platforms were categorized as digital vs traditional, 450 (75%) participants selected digital channels (WhatsApp, other chat platforms, or a customized PrEP app) as their most preferred platform for receiving PrEP support, while only 150 (25%) participants ranked traditional channels (SMS text messaging or phone calls) as their top choice.

Table 1 provides sociodemographic and behavioral characteristics by preferred communication channel category for PrEP support. Compared to participants who preferred traditional channels, those who preferred digital channels were more likely to reside in Metropolitan Lima (352/450, 78.2% vs 98/150, 65.3%; *P*=.002) and to report having more than 5 anal sex partners in the past 6 months (137/450, 30.4% vs 32/150, 21.3%; *P*=.03). Participants preferring digital channels were also significantly more likely to own a smartphone (448/450, 99.6% vs 141/150, 94.0%; *P*<.001). Other characteristics, including age, income, education level, recruitment method, perceived HIV risk, STI history, and condom use, did not differ significantly between groups.

**Table 1.** Sociodemographic and behavioral characteristics of HIV-negative men who have sex with men participating in a cross-sectional online survey assessing communication technology use and preferences for mHealth^a^ features to support HIV PrEP^b^ engagement in Peru (June 16-August 31, 2023; N=600), stratified by preferred communication channel for PrEP support.

Characteristics	Overall (N=600), n (%)	Traditional channels^c^ (n=150), n (%)	Digital channels^d^ (n=450), n (%)	*P* value^e^
Age (years)				.12
<25	152 (25.3)	32 (21.3)	120 (26.7)	
≥25	448 (74.7)	118 (78.7)	330 (73.3)	
Monthly income (PEN)^f^				.65
<1000	197 (32.8)	47 (31.3)	150 (33.3)	
≥1000	403 (67.2)	103 (68.7)	300 (66.7)	
Educational level				.77
Elementary or secondary	121 (20.2)	29 (19.3)	92 (20.4)	
Postsecondary	479 (79.8)	121 (80.7)	358 (79.6)	
Location				.002
Metropolitan Lima	450 (75)	98 (65.3)	352 (78.2)	
All other cities	150 (25)	52 (34.7)	98 (21.8)	
Recruitment method				.06
Organic content^g^ (unpaid)	305 (50.8)	64 (42.7)	241 (53.6)	
Instagram and Facebook ads	216 (36)	61 (40.7)	155 (34.4)	
Grindr ads	79 (13.2)	25 (16.7)	54 (12)	
Owns a smartphone				<.001
No	11 (1.8)	9 (6)	2 (0.4)	
Yes	589 (98.2)	141 (94.0)	448 (99.6)	
Perceived HIV risk^h^				.20
No	160 (26.7)	46 (30.7)	114 (25.3)	
Yes	440 (73.3)	104 (69.3)	336 (74.7)	
Engaged in anal sex^h^				.64
No	124 (20.7)	33 (22.0)	91 (20.2)	
Yes	476 (79.3)	117 (78.0)	359 (79.8)	
Anal sex partners^h^				.03
≤5 partners	431 (71.8)	118 (78.7)	313 (69.6)	
>5 partners	169 (28.2)	32 (21.3)	137 (30.4)	
Condom use^h^				.15
Always	246 (41)	69 (46)	177 (39.3)	
Not always	354 (59)	81 (54)	273 (60.7)	
Transactional sex^h^				.08
No	505 (84.2)	133 (88.7)	372 (82.7)	
Yes	95 (15.8)	17 (11.3)	78 (17.3)	
Recent STI^i^ diagnosis^h^				.83
No	432 (72)	107 (71.3)	325 (72.2)	
Yes	168 (28)	43 (28.7)	125 (27.8)	

a mHealth: mobile health

b PrEP: preexposure prophylaxis.

c Includes phone calls and SMS text messaging.

d Includes WhatsApp, a customized PrEP app, and other messaging platforms (eg, Telegram, Messenger, Instagram, TikTok).

e *P* values correspond to chi-square tests, except where expected cell counts were <5, in which case Fisher exact test was used.

f PEN: Peruvian soles (1000 PEN approximately US $268 as of August 15, 2023).

g Organic content refers to standard posts shared via the participating HIV and AIDS service organizations’ social media accounts and WhatsApp groups for study recruitment, without paid promotion.

h Behavioral variables refer to the past 6 months.

i STI: sexually transmitted infection (chlamydia, gonorrhea, or syphilis).

### Preferences for mHealth Tool Features for PrEP Support

**Figure 3.**
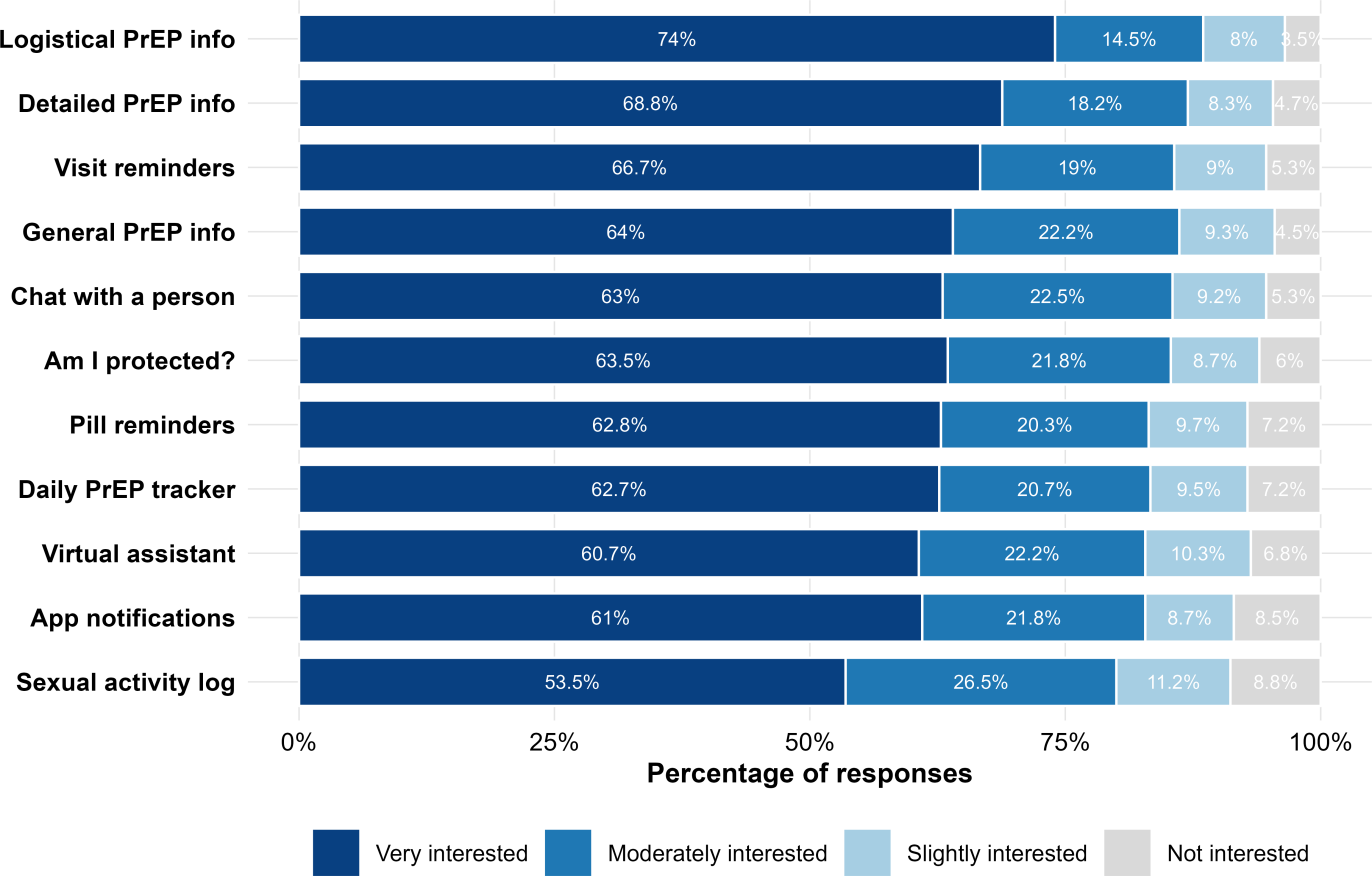
Distribution of participant-reported interest in specific mobile health features to support HIV preexposure prophylaxis use among HIV-negative men who have sex with men in Peru (cross-sectional online survey, June 16-August 31, 2023; N=600). Bars represent the proportion of respondents selecting each Likert-scale category (not interested, slightly interested, moderately interested, and very interested), and segments sum to 100% per feature. PrEP: preexposure prophylaxis.

Overall, interest was high across mHealth features for PrEP support (Figure 3 and Figures S4 and S5 in Multimedia Appendix 2). The most frequently endorsed feature was logistical information, such as the locations where PrEP is offered, clinic hours, frequency of visits, and cost, with 444 (74%) reporting they were “very interested.” This was followed by detailed PrEP information, including whether someone is a good candidate, possible side effects, and the steps required to initiate PrEP (413/600, 68.8%), appointment reminders (400/600, 66.7%), and general PrEP information, such as what PrEP is, how it works, and how to take it (384/600, 64%). Features such as chatting with a person (378/600, 63%), pill reminders (377/600, 62.8%), a daily PrEP tracker (376/600, 62.7%), and a virtual assistant (364/600, 60.7%) also generated strong interest. App notifications (366/600, 61%) and the “Am I protected?” feature (381/600, 63.5%) were similarly well rated. The sexual activity log received the lowest endorsement, though over half of participants (321/600, 53.5%) still indicated they were “very interested.”

### EFA of mHealth Tool Features for PrEP Support

The Kaiser-Meyer-Olkin measure indicated excellent sampling adequacy (0.93), and the Bartlett test of sphericity was significant (*χ*²_55_=7582.29; *P*<.001), supporting the suitability of the data for factor analysis. The EFA of the 11 items yielded a 3-factor solution explaining 81% of total variance and demonstrating high internal consistency for each factor (Table 2). The three domains were interpreted as:

1. Informational support (Cronbach α=0.94) included features providing educational content and logistical guidance related to PrEP. This factor encompassed general PrEP information, more detailed content about candidacy and side effects, logistical details such as service locations and costs, and visit reminders.
2. Self-management tools (Cronbach α=0.94) captured interest in tools for daily adherence and self-monitoring. It included pill reminders, app notifications, a daily PrEP tracker, a sexual activity log, and the “Am I protected?” feature.
3. Interactive communication (Cronbach α=0.91) reflected a preference for real-time, 2-way support, whether through chatting with a person or a virtual assistant.

**Table 2.** Exploratory factor analysis^a^ of participant-reported interest in mHealth^b^ features to support HIV PrEP^c^ use among HIV-negative men who have sex with men in Peru (cross-sectional online survey, June 16-August 31, 2023; N=600).

Items	Self-management tools (Cronbach α=0.94), factor loadings	Informational support (Cronbach α=0.94), factor loadings	Interactive communication (Cronbach α=0.91), factor loadings
General information about PrEP (eg, how it works, who it is intended for)	—^d^	0.80	—
Detailed medical information about PrEP (eg, side effects, lab tests, and dosing)	—	0.95	—
Logistical information on where and how to get PrEP (eg, nearby clinics, schedules, and costs)	—	0.90	—
Reminders for upcoming clinic appointments	—	0.63	—
Daily pill-taking reminders or dosing alerts	0.51	—	—
Push notifications from the app with updates or support	0.94	—	—
Daily tracker to monitor PrEP use	0.92	—	—
Log to record recent sexual activity (number of partners, condom use, etc)	0.74	—	—
Feature estimating HIV protection based on reported pill use and sexual activity	0.65	—	—
Virtual assistant (chatbot) providing automatic responses	—	—	0.54
Option to chat with a real person for support or questions	—	—	0.96

a Principal axis factoring with Promax (oblique) rotation. All retained factors had eigenvalues >1.0 and were supported by scree plot and parallel analysis. No substantial cross-loadings (>0.40 on more than one factor) were observed.

b mHealth: mobile health.

c PrEP: preexposure prophylaxis.

d Factor loadings <0.40 are not displayed for clarity.

Median scores were skewed toward the upper end of the scale, reflecting generally high levels of interest across all mHealth domains, with scores of 4.00 (IQR 3.00‐4.00) for informational support and interactive communication, and 3.80 (IQR 3.00‐4.00) for self-management tools. Even among participants who were unsure or not interested in PrEP at the time of the survey, interest in mHealth tools remained moderate to high, with median scores of 3.00 (IQR 2.00‐3.50) for informational support, 2.70 (IQR 1.80‐3.60) for self-management tools, and 3.00 (IQR 2.00‐4.00) for interactive communication. As expected, interest was significantly higher among those who were currently interested in using PrEP, with median scores of 4.00 (IQR 3.50‐4.00), 3.80 (IQR 3.00‐4.00), and 4.00 (IQR 3.00‐4.00) across the respective domains (all *P*<.001).

### Association Between Interest in mHealth Tool Features for PrEP Support and PrEP Cascade Stages

#### Decided to Use PrEP

Participants who had decided to use PrEP (483/600, 80.5%) were more likely than those who were unsure or had not decided (117/600, 19.5%) to reside in Metropolitan Lima (375/483, 77.6% vs 75/117, 64.1%; *P*=.002), perceive themselves at risk for HIV (382/483, 79.1% vs 58/117, 49.6%; *P*<.001), report anal sex in the past 6 months (392/483, 81.2% vs 84/117, 71.8%; *P*=.03), and have more than 5 anal sex partners (148/483, 30.6% vs 21/117, 17.9%; *P*=.006). In adjusted analyses, greater interest in informational support (aOR 4.54, 95% CI 3.36‐6.28; *P*<.001), self-management tools (aOR 3.23, 95% CI 2.51‐4.22; *P*<.001), and interactive communication (aOR 2.74, 95% CI 2.15‐3.53; *P*<.001) was associated with higher odds of having decided to use PrEP. Full results are provided in Table 3.

**Table 3.** Associations between interest in mHealth^a^ feature domains and decision to use HIV PrEP^b^ among HIV-negative men who have sex with men in Peru (cross-sectional online survey, June 16-August 31, 2023; N=600).

Characteristics	Decided to use PrEP^c^		Bivariate	Multivariable^d^
	Exposed, n/N (%)	Reference, n/N (%)	OR^e^ (95% CI)	aOR^f^ (95% CI)
Age ≥25 years	366/448 (81.7)	117/152 (77.0)	1.34 (0.85‐2.07)	—^g^
Postsecondary education	389/479 (81.2)	94/121 (77.7)	1.24 (0.75‐2.00)	—
Income >1000 PEN^h^	330/403 (81.9)	153/197 (77.7)	1.30 (0.85‐1.97)	—
Resides in Metropolitan Lima	375/450 (83.3)	108/150 (72.0)	1.96 (1.25‐3.03)^i^	1.92 (1.20‐3.03)^i^
Self-perceived HIV risk^j^	382/440 (86.8)	101/160 (63.1)	3.85 (2.52‐5.89)^k^	3.64 (2.36‐5.63)^k^
Engaged in anal sex^j^	392/476 (82.4)	91/124 (73.4)	1.69 (1.06‐2.67)^l^	—
Recent STI^m^ diagnosis	138/168 (82.1)	345/432 (79.9)	1.16 (0.74‐1.86)	—
>5 anal sex partners^j^	148/169 (87.6)	335/431 (77.7)	2.02 (1.23‐3.44)^i^	1.54 (0.92‐2.68)
Condomless sex^j^	293/354 (82.8)	190/246 (77.2)	1.42 (0.94‐2.13)	—
Transactional sex^j^	82/95 (86.3)	401/505 (79.4)	1.64 (0.90‐3.18)	—
mHealth features^n^				
Informational support^o^	2.75 (2.00‐3.50)^p^	4.00 (3.75‐4.00)^q^	4.99 (3.73‐6.85)^k^	4.54 (3.36‐6.28)^k^
Self-management tools^o^	2.80 (1.80‐3.60)^p^	4.00 (3.20‐4.00)^q^	3.53 (2.76‐4.59)^k^	3.23 (2.51‐4.22)^k^
Interactive communication^o^	3.00 (2.00‐3.50)^p^	4.00 (3.00‐4.00)^q^	2.97 (2.36‐3.78)^k^	2.74 (2.15‐3.53)^k^

a mHealth: mobile health.

b PrEP: preexposure prophylaxis.

c Exposed n/N (%) and reference n/N (%) indicate the number and proportion of participants with the outcome (decided to use PrEP) within each exposure category.

d Multivariable models were built using a two-step approach. First, stepwise selection was applied to all sociodemographic and behavioral variables to identify covariates independently associated with the decision to use PrEP. Second, each mHealth factor score was entered into a separate multivariable logistic regression model, adjusted for the covariates selected in step one. Variance inflation factors (VIFs) confirmed no multicollinearity (all <2).

e OR: odds ratio.

f aOR: adjusted odds ratio.

g Not available.

h PEN: Peruvian soles (1000 PEN approximately US $268 as of August 15, 2023).

i *P*<.01.

j Behavioral variables refer to the past 6 months.

k *P*<.001.

l *P*<.05.

m STI: sexually transmitted infection (chlamydia, gonorrhea, or syphilis).

n mHealth domain scores were derived from exploratory factor analysis using principal axis factoring with Promax rotation. Scores are presented as median (IQR) and were entered as continuous variables in regression models.

o Multivariable models adjusted for city of residence, self-perceived HIV risk, and number of anal sex partners.

p mHealth domain score among participants with the outcome (decided to use PrEP); median (IQR)

q mHealth domain score among participants without the outcome (decided to use PrEP); median (IQR)

#### Sought PrEP

Participants who had sought PrEP (190/600, 31.7%) were more likely than those who had not (410/600, 68.3%) to be aged 25 years or older (158/190, 83.2% vs 290/410, 70.7%; *P*=.001), have postsecondary education (161/190, 84.7% vs 318/410, 77.6%; *P*=.04), reside in Metropolitan Lima (161/190, 84.7% vs 289/410, 70.5%; *P*<.001), report condomless sex (124/190, 65.3% vs 230/410, 56.1%; *P*=.03), have more than 5 anal sex partners (72/190, 37.9% vs 97/410, 23.7%; *P*<.001), engage in transactional sex (41/190, 21.6% vs 54/410, 13.2%; *P*=.009), and report a recent STI diagnosis (68/190, 35.8% vs 100/410, 24.4%; *P*=.004). In adjusted analyses, higher interest in informational support (aOR 1.43, 95% CI 1.10‐1.89; *P*=.001) and self-management tools (aOR 1.34, 95% CI 1.06‐1.70; *P*=.02) was associated with having sought PrEP, whereas the association with interactive communication was marginal (aOR 1.25, 95% CI 1.00‐1.57; *P*=.053). Full results are provided in Table 4.

**Table 4.** Associations between interest in mHealth^a^ feature domains and having sought HIV PrEP^b^ among HIV-negative men who have sex with men in Peru (cross-sectional online survey, June 16-August 31, 2023; N=600).

Characteristics	Sought PrEP^c^		Bivariate	Multivariable^d^
	Exposed, n/N (%)	Reference, n/N (%)	OR^e^ (95% CI)	aOR^f^ (95% CI)
Age ≥25 years	158/448 (35.3)	32/152 (21.1)	2.04 (1.34‐3.20)^g^	1.74 (1.09‐2.82)^h^
Postsecondary education	161/479 (33.6)	29/121 (24.0)	1.61 (1.03‐2.58)^h^	1.49 (0.91‐2.50)
Income >1000 PEN^i^	136/403 (33.7)	54/197 (27.4)	1.35 (0.93‐1.97)	—^j^
Resides in Metropolitan Lima	161/450 (35.8)	29/150 (19.3)	2.33 (1.49‐3.70)^k^	2.22 (1.41‐3.57)^k^
Self-perceived HIV risk^l^	149/440 (33.9)	41/160 (25.6)	1.49 (1.00‐2.25)	—
Engaged in anal sex^l^	153/476 (32.1)	37/124 (29.8)	1.11 (0.73‐1.73)	—
Recent STI^m^ diagnosis^l^	68/168 (40.5)	122/432 (28.2)	1.73 (1.19‐2.51)^g^	1.47 (0.99‐2.18)
>5 anal sex partners^l^	72/169 (42.6)	118/431 (27.4)	1.97 (1.36‐2.85)^k^	1.62 (1.09‐2.41)^h^
Condomless sex^l^	124/354 (35.0)	66/246 (26.8)	1.47 (1.03‐2.11)^h^	—
Transactional sex^l^	41/95 (43.2)	149/505 (29.5)	1.81 (1.15‐2.84)^g^	1.63 (1.01‐2.62)^h^
mHealth features^n^				
Informational support^o^	4.00 (3.00‐4.00)^p^	4.00 (3.50‐4.00)^q^	1.54 (1.20‐2.03)^g^	1.43 (1.10‐1.89)^h^
Self-management tools^o^	3.60 (3.00‐4.00)^p^	4.00 (3.20‐4.00)^q^	1.42 (1.14‐1.80)^g^	1.34 (1.06‐1.70)^h^
Interactive communication^o^	4.00 (3.00‐4.00)^p^	4.00 (3.00‐4.00)^q^	1.32 (1.07‐1.65)^h^	1.25 (1.00‐1.57)

a mHealth: mobile health.

b PrEP: preexposure prophylaxis.

c Exposed n/N (%) and reference n/N (%) indicate the number and proportion of participants with the outcome (sought PrEP) within each exposure category.

d Multivariable models were built using a two-step approach. First, stepwise selection was applied to all sociodemographic and behavioral variables to identify covariates independently associated with having sought PrEP. Second, each mHealth factor score was entered into a separate multivariable logistic regression model, adjusted for the covariates selected in step one. Variance inflation factors (VIFs) confirmed no multicollinearity (all <2).

e OR: odds ratio.

f aOR: adjusted odds ratio.

g *P*<.01

h *P*<.05.

i PEN: Peruvian soles (1000 PEN: approximately US $268 as of August 15, 2023).

j Not available.

k *P*<.001

l Behavioral variables refer to the past 6 months.

m STI: sexually transmitted infection (chlamydia, gonorrhea, or syphilis).

n mHealth domain scores were derived from exploratory factor analysis using principal axis factoring with Promax rotation. Scores are presented as median (IQR) and were entered as continuous variables in regression models.

o Multivariable models adjusted for age, education, city of residence, recent STI diagnosis, number of anal sex partners, and transactional sex.

p mHealth domain score among participants with the outcome (sought PrEP); median (IQR).

q mHealth domain score among participants without the outcome (sought PrEP); median (IQR).

#### PrEP Initiation

Participants who had initiated PrEP (109/600, 18.2%) were more likely than those who had not (491/600, 81.8%) to report a monthly income above 1000 PEN (87/109, 79.8% vs 316/491, 64.4%; *P*=.002), have postsecondary education (96/109, 88.1% vs 383/491, 78%; *P*=.02), reside in Metropolitan Lima (94/109, 86.2% vs 356/491, 72.5%; *P*=.003), and report a recent STI diagnosis (42/109, 38.5% vs 126/491, 25.7%; *P*=.007). In adjusted analyses, higher interest in informational support (aOR 1.64, 95% CI 1.16‐2.44; *P*=.009) and self-management tools (aOR 1.49, 95% CI 1.11‐2.05; *P*=.01) was associated with PrEP initiation, whereas interactive communication was not significantly associated. Full results are provided in Table 5.

**Table 5.** Associations between interest in mHealth^a^ feature domains and initiating HIV PrEP^b^ among HIV-negative men who have sex with men in Peru (cross-sectional online survey, June 16-August 31, 2023; N=600).

Characteristics	Ever initiated PrEP^c^		Bivariate	Multivariable^d^
	Exposed, n/N (%)	Reference, n/N (%)	OR^e^ (95% CI)	aOR^f^ (95% CI)
Age ≥25 years	88/448 (19.6)	21/152 (13.8)	1.52 (0.93‐2.61)	—^g^
Postsecondary education	96/479 (20.0)	13/121 (10.7)	2.08 (1.16‐4.03)^h^	1.78 (0.95‐3.55)
Income >1000 PEN^i^	87/403 (21.6)	22/197 (11.2)	2.19 (1.35‐3.70)^j^	1.85 (1.10‐3.22)^h^
Resides in Metropolitan Lima	94/450 (20.9)	15/150 (10.0)	2.38 (1.37‐4.35)^j^	2.27 (1.28‐4.17)^j^
Self-perceived HIV risk^k^	85/440 (19.3)	24/160 (15.0)	1.36 (0.84‐2.26)	—
Engaged in anal sex^k^	83/476 (17.4)	26/124 (21.0)	0.80 (0.49‐1.32)	—
Recent STI^l^ diagnosis^k^	42/168 (25.0)	67/432 (15.5)	1.82 (1.17‐2.80)^j^	1.83 (1.17‐2.86)^j^
>5 anal sex partners^k^	39/169 (23.1)	70/431 (16.2)	1.55 (0.99‐2.39)	—
Condomless sex^k^	67/354 (18.9)	42/246 (17.1)	1.13 (0.74‐1.74)	—
Transactional sex^k^	18/95 (18.9)	91/505 (18.0)	1.06 (0.59‐1.83)	—
mHealth features^m^				
Informational support^n^	4.00 (3.00‐4.00)^o^	4.00 (3.75‐4.00)^p^	1.76 (1.25‐2.60)^j^	1.64 (1.16‐2.44)^j^
Self-management tools^n^	3.60 (3.00‐4.00)^o^	4.00 (3.40‐4.00)^p^	1.57 (1.17‐2.16)^j^	1.49 (1.11‐2.05)^h^
Interactive communication^n^	4.00 (3.00‐4.00)^o^	4.00 (3.00‐4.00)^p^	1.25 (0.97‐1.66)	1.20 (0.93‐1.60)

a mHealth: mobile health.

b PrEP: preexposure prophylaxis.

c Exposed n/N (%) and reference n/N (%) indicate the number and proportion of participants with the outcome (initiated PrEP) within each exposure category.

d Multivariable models were built using a two-step approach. First, stepwise selection was applied to all sociodemographic and behavioral variables to identify covariates independently associated with PrEP initiation. Second, each mHealth factor score was entered into a separate multivariable logistic regression model, adjusted for the covariates selected in step one. Variance inflation factors (VIFs) confirmed no multicollinearity (all <2).

e OR: odds ratio.

f aOR: adjusted odds ratio.

g Not available.

h *P*<.05.

i PEN: Peruvian soles (1000 PEN: approximately US $268 as of August 15, 2023).

j *P*<.01.

k Behavioral variables refer to the past 6 months.

l STI: sexually transmitted infection (chlamydia, gonorrhea, or syphilis).

m mHealth domain scores were derived from exploratory factor analysis using principal axis factoring with Promax rotation. Scores are presented as median (IQR) and were entered as continuous variables in regression models.

n Multivariable models adjusted for education, income, city of residence and recent STI diagnosis.

o mHealth domain score among participants with the outcome (initiated PrEP); median (IQR).

p mHealth domain score among participants with the outcome (initiated PrEP); median (IQR).

#### Multinomial Sensitivity Analyses

As a sensitivity analysis, we fit multinomial logistic regression models treating PrEP cascade stage as a 4-level categorical outcome (not decided, decided only, sought PrEP, and initiated PrEP), using “not decided” as the reference category. The distribution of cascade stages was as follows: not decided (101/600, 16.8%), decided only (285/600, 47.5%), sought PrEP only (105/600, 17.5%), and initiated PrEP (109/600, 18.2%). Associations were broadly consistent with the stage-specific logistic regression models (Table 6).

**Table 6.** Multinomial analysis^a^ of associations between interest in mHealth^b^ feature domains and engagement across stages of the HIV PrEP^c^ cascade among HIV-negative men who have sex with men in Peru (cross-sectional online survey, June 16-August 31, 2023; N=600).

Multinomial analysis	Decided only vs not decided, aRRR^d^ (95% CI)	Sought PrEP vs not decided, aRRR (95% CI)	Initiated PrEP vs not decided, aRRR (95% CI)
PrEP cascade stage vs not decided (each mHealth domain entered separately)			
Score: informational support^e^	4.18 (2.97‐5.87)^f^	3.02 (2.04‐4.49)^f^	4.18 (2.65‐6.57)^f^
Score: self-management tools^e^	3.25 (2.42‐4.35)^f^	2.57 (1.82‐3.64)^f^	3.31 (2.27‐4.83)^f^
Score: interactive support^e^	2.93 (2.20‐3.89)^f^	2.26 (1.62‐3.17)^f^	2.45 (1.74‐3.44)^f^
All mHealth scores in one model (mutually adjusted)			
Score: informational support^e^	2.99 (1.67‐5.34)^f^	2.44 (1.22‐4.89)^g^	3.37 (1.58‐7.19)^h^
Score: self-management tools^e^	1.49 (0.84‐2.64)	1.50 (0.76‐2.95)	2.03 (0.99‐4.16)
Score: interactive support^e^	0.98 (0.57‐1.70)	0.85 (0.45‐1.60)	0.63 (0.33‐1.20)

a Multinomial models were adjusted for prespecified sociodemographic and behavioral covariates (age category, income, education, location, perceived HIV risk, number of anal sex partners, recent STI diagnosis, and transactional sex) included simultaneously in all models. Models in the upper panel include each mHealth domain score entered separately. Models in the lower panel include all three mHealth domain scores simultaneously (mutually adjusted).

b mHealth: mobile health.

c PrEP: preexposure prophylaxis.

d aRRR: adjusted relative risk ratio.

e mHealth domain scores were derived from exploratory factor analysis using principal axis factoring with Promax rotation and entered as continuous variables in regression models.

f *P*<.001.

g *P*<.05.

h *P*<.01.

In models including each mHealth domain separately, higher scores were strongly associated with engagement in more advanced cascade stages compared with not having decided to use PrEP. For example, for each 1-unit increase in the informational support score, the relative risk of being in the decided-only, sought, and initiated stages was approximately 3- to 4-fold higher compared with the not-decided stage, with similar magnitudes observed for the self-management tools and interactive support scores. When all 3 domain scores were included simultaneously, the informational support score remained independently associated with more advanced cascade stages relative to the not-decided stage, whereas associations for the self-management tools and interactive support scores were attenuated, suggesting shared variance across domains. When cascade stages were rereferenced to examine adjacent contrasts, domain scores were strongly associated with the contrast between decided-only and not decided but were not significantly associated with transitions from decided-only to sought PrEP or from sought PrEP to initiated PrEP.

## Discussion

### Principal Findings

In this cross-sectional survey of HIV-negative MSM in Peru, we found near-universal smartphone ownership and a strong preference for digital communication platforms, particularly WhatsApp, for receiving PrEP-related support. Interest in informational support, self-management tools, and interactive communication features was high overall. Greater interest in these mHealth domains was associated with engagement across the PrEP cascade. Informational support and self-management features demonstrated consistent associations with all 3 stages, whereas interactive communication features were associated only with the decision stage. Associations were strongest for the decision stage and more modest for later stages. Sensitivity analyses using multinomial models showed similar overall patterns.

These findings are consistent with prior evidence suggesting that mHealth interventions can support engagement with HIV prevention services. A recent meta-analysis of randomized controlled trials reported that digital interventions improve HIV testing and PrEP adherence and show emerging effects on PrEP uptake [42]. Similarly, a systematic review among MSM concluded that digital tools are generally feasible and acceptable and may support engagement across the PrEP continuum when culturally tailored and user-centered [43]. Together, this body of evidence underscores the potential role of mHealth as an entry point into prevention services that can be integrated with offline initiation and follow-up within differentiated service delivery models [24,44]. Our findings extend this literature by identifying which types of digital support may be most salient at different stages of PrEP engagement. In particular, the stage-specific associations observed in our analyses provide insight into when digital supports may be most useful within the PrEP cascade.

The stronger associations at the decision stage, compared with the more modest associations at later stages, suggest that digital features may be particularly salient during early phases of PrEP engagement. At these stages, individuals may be evaluating their HIV risk, learning about prevention options, and determining whether and how to access services. Digital tools delivered through widely used communication platforms could therefore help translate risk awareness into concrete prevention decisions by providing accessible information about eligibility, service availability, and pathways to PrEP initiation. In contrast, progression through later cascade stages, such as seeking services and initiating PrEP, may depend more heavily on structural and health system factors, including service accessibility, appointment availability, and provider interactions.

Informational support demonstrated the strongest and most consistent associations across the PrEP cascade stages examined in this study, underscoring the continued importance of accurate, accessible, and actionable information about PrEP. Features that clarify where and how to obtain PrEP, including service locations, clinic hours, costs, and eligibility criteria, may be particularly relevant in contexts where program expansion is ongoing. Despite expansion of PrEP services in Peru, uncertainty about access pathways may persist. Digital informational tools may help address these barriers by centralizing prevention information and simplifying service navigation. For example, decision-support and navigation platforms such as MyPrEP [26], SaludFindr [31], and PrEPresent [25] have been designed to help users assess PrEP eligibility, locate services, and connect with prevention programs. Clinic-integrated platforms such as JomPrEP [23,24] extend these functions by combining informational resources with service navigation features, including HIV self-testing support, teleconsultation, and streamlined pathways for PrEP initiation, within a mobile interface. In the Peruvian context, similar tools could be adapted to incorporate location-specific service directories, appointment scheduling, and referral pathways. Emerging approaches, including the integration of generative artificial intelligence to dynamically update service directories, eligibility guidance, and prevention information, may further enhance scalability and timeliness of such tools [45-47], although their effectiveness in real-world PrEP delivery settings warrants evaluation.

Likewise, self-management tools including pill reminders, adherence trackers, protection status calculators, and sexual activity logs were also associated with greater engagement across the PrEP cascade, suggesting that users value tools that support adherence, routine formation, and ongoing self-monitoring. These functionalities are designed to support adherence and ongoing engagement after initiation, and evidence from mobile interventions in other settings suggests they can be feasible and acceptable to users. For example, tools such as PrEPmate [15], iTAB [16], the Amsterdam PrEP app [17], PrEP-iT! [18], P3 [19], and DOT Diary [20] incorporate adherence reminders, monitoring systems, or behavioral feedback features and have demonstrated high usability and feasibility, with some studies reporting improvements in PrEP adherence. In the Peruvian context, similar tools could be adapted with appropriate cultural and linguistic tailoring. Core features such as reminders and simplified adherence tracking could also be integrated into widely used messaging platforms. Emerging digital approaches may facilitate scalable adaptation; however, their effectiveness and implementation feasibility should be evaluated within local health system contexts.

By contrast, interactive communication features appeared to be most relevant earlier in the cascade, with the strongest association observed for the decision to use PrEP. This pattern suggests that personalized, bidirectional communication may support individuals as they explore PrEP and clarify uncertainties. Digital conversational tools, including AI-enabled chatbots, have been proposed as scalable mechanisms to deliver tailored prevention information and facilitate service navigation while operating continuously and triaging more complex inquiries to human providers [48]. Early implementations of chatbot-based HIV prevention tools such as Amanda Selfie [27] and CHIA [30] have demonstrated feasibility and user acceptability in pilot studies. MYHIV365, a chatbot-based digital intervention developed in Malaysia to support HIV prevention among MSM, integrates conversational guidance on HIV testing, PrEP awareness, and risk reduction with culturally tailored content designed to address stigma and informational barriers [28,29]. Early evaluations have reported high usability and acceptability, suggesting that conversational agents may provide a scalable mechanism to deliver prevention information and support decision-making in stigmatized or resource-constrained settings.

Because such conversational systems can be deployed through widely used messaging platforms such as WhatsApp, they may offer a practical approach to delivering interactive prevention support within existing communication ecosystems. The widespread and frequent use of WhatsApp observed in our sample further supports the feasibility of this approach. Nearly all participants owned smartphones and reported frequent use of WhatsApp for daily communication, consistent with prior reports documenting the central role of messaging platforms in digital health engagement across diverse settings [8,28,49-52]. In contexts such as Peru, where developing and maintaining dedicated health applications may be resource-intensive for public health systems, leveraging existing communication platforms may offer a pragmatic strategy for delivering scalable interactive prevention support. Earlier work among MSM in Lima reported substantially lower smartphone access in 2015 [7], suggesting that the rapid expansion of digital connectivity over the past decade may enable broader implementation of messaging-based prevention tools. As conversational technologies evolve, integrating AI-enabled chatbots with clinical messaging workflows may provide scalable models for delivering personalized PrEP guidance and follow-up within routine prevention services.

The potential value of these digital supports will likely depend on how well they align with local communication habits, user preferences, social norms, health system capacity, and service pathways. Adapting mHealth tools for the Peruvian context by embedding local clinical pathways, incorporating culturally and linguistically appropriate content, and integrating with widely used platforms may enhance their relevance across different stages of the PrEP cascade. Prior Spanish-language adaptations of digital HIV interventions have demonstrated the feasibility of this approach and underscore the importance of aligning tools with local communication norms and service structures [31,32]. Together, these considerations support the development of contextually grounded digital strategies rather than the direct transplantation of tools developed for other health system environments.

In addition to linguistic and service-delivery adaptation, digital tools may also be relevant because they can address barriers related to privacy and anticipated stigma. Although stigma was not directly measured in this study, prior research in Peru indicates that discrimination and discomfort in health care settings remain important barriers to PrEP uptake [53-55]. Digital support tools have been proposed as an implementation strategy to reduce structural and interpersonal barriers by allowing individuals to access information and decision support more privately [12,56]. By offering discreet engagement with credible sources and supportive communication, mHealth approaches may lower some perceived barriers to initial PrEP exploration. Digital support strategies should be viewed as complementary to, rather than replacements for, broader efforts to address stigma within health care systems.

Beyond individual feature preferences, our findings also have implications for how digital supports might be embedded within broader PrEP delivery models. Two relevant approaches are online-to-offline linkage models and telePrEP. The high level of interest in digital support features observed in this study is consistent with the potential relevance of online-to-offline approaches for PrEP delivery. In these models, individuals are initially engaged through digital platforms such as targeted messaging, educational content, or virtual navigation, and subsequently linked to in-person services through facilitated referrals, electronic appointment systems, or digital vouchers [57]. Such models may be particularly pertinent in settings where the geographic concentration of services and perceived stigma create barriers to in-person engagement [58,59]. The Adam’s Love online-to-offline model in Thailand, which increased HIV testing and PrEP uptake through integrated online counseling and streamlined clinic check-in processes, illustrates how digital engagement can be operationalized within routine service delivery [60]. Adaptation of similar models to the Peruvian context would require integration with local service pathways and evaluation of their feasibility and impact.

TelePrEP represents a complementary strategy within differentiated service delivery models [56]. By incorporating virtual consultations, remote prescribing, and home-based monitoring, telePrEP has been proposed as a mechanism to expand access for individuals who prefer remote care or who reside in areas with limited clinic availability [61]. Integration of mHealth features such as messaging-based reminders, adherence tracking, and location-specific informational support into telePrEP platforms may facilitate continuity between digital engagement and clinical services. In combination, online-to-offline approaches and telePrEP models illustrate how digital strategies could be configured to support more flexible and geographically inclusive PrEP delivery frameworks in Peru. Formal evaluation would be needed to determine their effectiveness and scalability within the national health system.

As access to long-acting PrEP expands, including twice-yearly lenacapavir, which has been approved in the United States and recommended by the World Health Organization as an additional PrEP option [62,63], priorities for digital support may shift toward earlier stages of the prevention cascade. Because twice-yearly injectable PrEP reduces daily adherence demands compared with oral regimens, digital tools may become increasingly relevant for awareness, risk appraisal, demand generation, and linkage to services, while still supporting safety monitoring and follow-up. Ongoing research is also evaluating lenacapavir formulations administered at extended intervals [64], which could further reduce adherence-related barriers and increase the relative importance of timely, localized information regarding eligibility, service availability, cost, and evolving guidance. Integration of these informational and navigation functions into widely used platforms such as WhatsApp may support engagement as long-acting options become available, while maintaining pathways to human support.

This study has several limitations. First, the cross-sectional online survey design likely oversampled individuals who are urban and digitally connected, which may limit generalizability to populations with more limited internet access and to other groups not included in this analysis. Second, all measures were self-reported, including behavioral indicators and interest in digital features, which may be subject to recall or reporting bias. Although anonymous online surveys may reduce social desirability bias [65], measurement error remains possible. Third, interest was assessed for hypothetical mHealth features rather than actual tool use or clinical outcomes and therefore observed associations with PrEP cascade stages should not be interpreted as evidence of effectiveness. Fourth, the cross-sectional design precludes causal inference regarding the directionality of associations. Finally, stigma was not directly measured and therefore could not be examined as a mediator or moderator of digital engagement preferences.

These limitations underscore the need for prospective implementation research to evaluate the feasibility, acceptability, and effectiveness of specific mHealth interventions within diverse Peruvian settings. Implementation science frameworks may provide structured guidance for adapting and evaluating digital tools in real-world contexts [51,66]. By identifying which domains of digital support are most strongly associated with engagement at different stages of the PrEP cascade, our findings offer practical guidance for designing user-centered digital strategies to strengthen HIV prevention delivery in Peru and similar settings.

### Conclusions

In summary, this study provides novel, stage-specific evidence on digital preferences that may support PrEP engagement in a Latin American implementation context. Informational support and self-management tools were consistently associated with engagement across multiple stages of the PrEP cascade, whereas interactive communication features appeared most relevant during early decision-making phases. Among these domains, informational support emerged as the most consistent correlate of reaching more advanced cascade stages, highlighting the importance of accessible and actionable information in facilitating progression along the prevention continuum. These findings extend the existing literature by identifying which digital functions may be most salient at different points in the prevention cascade.

From a public health perspective, the near-universal smartphone ownership and strong preference for WhatsApp suggest that culturally tailored, messaging-based digital strategies aligned with specific cascade stages could be feasibly integrated within differentiated service delivery models, including telePrEP and online-to-offline frameworks. As long-acting PrEP options expand and delivery models evolve, contextually adapted mHealth tools may help align prevention services with users’ communication practices and complement efforts to address structural barriers to engagement. Rigorous implementation research is needed to determine their effectiveness, scalability, and sustainability within Peru’s national PrEP program and similar health system environments.

## Supplementary material

10.2196/84982 Multimedia Appendix 1 Mobile health survey in Spanish.

10.2196/84982 Multimedia Appendix 2 Additional tables and figures.

## Abbreviations

aOR: adjusted odds ratio
EFA: exploratory factor analysis
mHealth: mobile health
MSM: men who have sex with men
PEN: Peruvian soles
PrEP: preexposure prophylaxis
STI: sexually transmitted infection
