# Supplementary material for: Preferences for mHealth Features to Support Engagement in the HIV Preexposure Prophylaxis Cascade Among Men Who Have Sex With Men in Peru: Cross-Sectional Online Survey
Source: JMIR Public Health Surveill. 2026 Jul 14;12:e84982. doi: 10.2196/84982 (PMC13367761; doi:10.2196/84982)
Supplement: Multimedia Appendix 1 [file publichealth-v12-e84982-s001.docx]

**Sección 1: Sociodemográficos**

1. Fecha de nacimiento (dd/mm/aaaa)

_____________________________

1. Sexo asignado al nacer
   1. Masculino
   2. Femenino
2. Género con el que te identificas.
   1. Hombre cisgénero
   2. Mujer cisgénero
   3. Mujer transgénero
   4. Hombre transgénero
   5. Otro
3. Eres una persona que vive con VIH?
   1. Si
   2. No
   3. No estoy seguro
4. ¿Cuál es tu ingreso mensual en promedio?
   1. < 1000 soles
   2. 1000-2000 soles
   3. 2000-3000 soles
   4. 3000-4000 soles
   5. >4000 soles
5. ¿Cuál es el máximo nivel educativo que has completado?
   1. Ninguno
   2. Primaria
   3. Secundaria
   4. Superior técnico
   5. Superior universitaria
6. ¿En dónde vives actualmente?
   Departamento:__________________________
   Provincia:_______________________________
   Distrito: ________________________________
7. ¿Has participado en alguno de los siguientes estudios de investigación?
   1. MOSAICO
   2. HPTN 083
   3. PrEP PERU
   4. ImPrEP
   5. Ninguno de los anteriores
   6. No estoy seguro

**Sección 2: Identificación del riesgo:**

1. Actualmente, ¿te consideras una persona en riesgo de adquirir VIH?
   1. Definitivamente no
   2. Probablemente no
   3. Probablemente si
   4. Definitivamente si
2. ¿Tienes sexo anal con hombres o mujeres transgénero?
   1. Si
   2. No
3. En los últimos 6 meses ¿Con cuántas personas has tenido sexo anal?
   1. Con ninguna
   2. Entre 1 y 5 personas
   3. Más de 5 personas
4. De las personas con las que has tenido sexo anal en los últimos 6 meses, ¿alguna tiene VIH?
   1. Definitivamente no
   2. No estoy seguro
   3. Si, pero al menos una de estas persona no está recibiendo tratamiento
   4. Si, pero todas estas personas están en tratamiento y son indetectables
5. Piensa en todas las veces que has tenido sexo anal en los últimos 6 meses. ¿Con qué frecuencia usaste condón?
   1. Siempre
   2. Casi siempre
   3. Algunas veces
   4. Casi nunca
   5. Nunca
6. En los últimos 6 meses, ¿has realizado trabajo sexual?
   1. Si
   2. No
   3. No estoy seguro
7. Cuéntanos si en los últimos 6 meses….

|  | Sí | No |
| --- | --- | --- |
| ¿Tuviste secreción o pus por el pene? |  |  |
| ¿Tuviste alguna úlcera o llaga en el pene o ano? |  |  |
| ¿Tuviste alguna verruga o carnosidad en el pene o ano? |  |  |
| ¿Tuviste secreción, pus o sangre por el ano? |  |  |
| ¿Tuviste algún diagnóstico de sífilis? |  |  |
| ¿Tuviste un hisopado positivo para gonorrea o clamidia? |  |  |

**Sección 3: PrEP y etapas de cambio**

*Ahora queremos hacerte unas preguntas sobre PrEP (Profilaxis PRE-exposición al VIH). Es posible que alguna vez hayas escuchado hablar sobre PrEP, incluso es posible que lo hayas usado o te encuentres actualmente usándolo. De todos modos, queremos que revises el siguiente cuadro que tiene información importante sobre el PrEP antes de responder las preguntas.*

1. ¿Te consideras actualmente un buen candidato para usar PrEP?
   1. Si
   2. No
   3. No estoy seguro
2. ¿Estarías interesado en tomar PrEP?
   1. Definitivamente tomaría PrEP
   2. Probablemente tomaría PrEP
   3. No estoy seguro si tomaría PrEP
   4. Probablemente no tomaría PrEP
   5. Definitivamente no tomaría PrEP
3. Para empezar PrEP se necesita una valuación y receta médicas. ¿Conoces algún consultorio, clínica, centro de salud u hospital dónde pudieras obtener una receta médica para PrEP?
   1. Si, y estoy seguro que en ese lugar dan recetas para PrEP
   2. Si, pero no estoy seguro si en ese lugar dan recetas para PrEP
   3. No
4. Actualmente, el estado ha empezado a brindar PrEP gratuitamente en algunos establecimientos de salud del primer nivel ¿Tienes decidido iniciar PrEP?
   1. Definitivamente empezaré a tomar PrEP
   2. Probablemente empezaré a tomar PrEP
   3. No estoy seguro si empezar a tomar PrEP
   4. Probablemente no empezaré a tomar PrEP
   5. Definitivamente no empezaré a tomar PrEP
5. ¿Alguna vez has conversado con un médico sobre iniciar PrEP?
   1. Si
   2. No
6. ¿Alguna vez has tomado PrEP?
   1. Si, actualmente estoy tomando
   2. Si, he usado pero no estoy usando actualmente
   3. No
7. En el último mes (30 días). ¿Cuántos días dejaste de tomar tu dosis de PrEP?
   1. _____________________
8. Las citas de PrEP son cada 3 meses ¿Estas acudiendo a tus visitas de PrEP de forma regular?
   1. Si
   2. No
   3. no estoy seguro

*También queremos hacerte unas preguntas sobre el uso de PEP (Profilaxis-POST Exposición al VIH). PEP es diferente a PrEP. Por favor, revisa la información del siguiente cuadro antes de responder las preguntas:*

1. En los últimos 6 meses. ¿Has solicitado PEP en algún establecimiento de salud por un contacto sexual de riesgo?
   1. Si
   2. No
2. ¿Por qué no solicitaste PEP?
   1. No considero haber tenido contactos sexuales de riesgo
   2. He tenido contactos de riesgo, pero no sabía que existía la PEP
   3. Sabía que exisitía la PEP, pero no sabía donde o cómo solicitarlo
   4. Sabía cómo y dónde obtener la PEP, pero no quise hacerlo
3. Cuando solicitaste PEP ¿Obtuviste las pastillas antes de que pasaran 72 horas del contacto de riesgo?
   1. Si
   2. No

**Sección 3: Procesos de cambio**

1. Cuéntanos lo que piensas sobre las siguientes afirmaciones respecto al uso de PrEP

|  | Totalmente de acuerdo | De acuerdo | En desacuerdo | Totalmente en desacuerdo |
| --- | --- | --- | --- | --- |
| Antes de hoy, ya había leído o escuchado que el PrEP puede protegerme del VIH |  |  |  |  |
| Antes de hoy, ya tenía conocimiento sobre cómo acceder al PrEP en el Perú |  |  |  |  |
| Conozco otras personas que están tomando PrEP |  |  |  |  |
| Creo que tengo suficiente información como para decidir o no iniciar PrEP |  |  |  |  |
| Pienso en que el PrEP puede ayudarme a estar protegido contra el VIH |  |  |  |  |
| He buscado información en internet o preguntado a otras personas sobre el PrEP |  |  |  |  |
| Me da miedo la idea de adquirir el VIH |  |  |  |  |
| Dejaría de tomar PrEP si me causara alguna molestia física aunque sea pasajera |  |  |  |  |
| Me sentiría menos preocupado del VIH usando PrEP |  |  |  |  |
| Pienso que usando PrEP también puedo proteger a mi(s) pareja(s) del VIH |  |  |  |  |
| Podría usar alarmas o recordatorios que me ayuden a tomar PrEP |  |  |  |  |
| Podría adaptar mi rutina diaria de forma que me facilite el tomar PrEP |  |  |  |  |
| Pienso que usar PrEP es algo actualmente aceptado por la sociedad |  |  |  |  |
| Mi(s) pareja(s) sexuales me agradecerían que yo usara PrEP |  |  |  |  |
| Me sentiría mejor conmigo mismo si tomara todos los días el PrEP |  |  |  |  |
| Las personas cercanas a mí me apoyarían en el uso de PrEP |  |  |  |  |
| Me preocupa el costo de las pastillas de PrEP |  |  |  |  |
| Me preocupa el costo de los exámenes médicos que se necesitan para tomar PrEP |  |  |  |  |
| Me siento capaz de tomar un pastilla diaria de PrEP |  |  |  |  |
| Me siento capaz de asistir a las citas para recibir las pastillas de PrEP |  |  |  |  |
| Pienso que tomar PrEP es bueno para mí, en lugar de ser una carga |  |  |  |  |
| Creo que podría tomar PrEP por largo tiempo |  |  |  |  |

1. En el momento actual, ¿Cómo ves los pros y contras de que empieces a tomar PrEP?
   1. Sólo veo contras
   2. Veo más contras que pros
   3. Los pros y contras son iguales
   4. Veo más pros que contras
   5. Sólo veo pros

**Sección 4: Uso de celulares para ayudar en el uso de PrEP**

*Ahora nos gustaría conocer lo que piensas sobre el uso de teléfonos celulares para recibir información o asistencia en temas de cuidados de la salud, como el uso de PrEP. Los teléfonos celulares pueden ayudar a que las personas reciban información, recordatorios o interactúen con el personal de salud sobre temas de cuidados de la salud, incluyendo el uso de PrEP.*

1. ¿Cuentas con un celular con internet?
   1. Si
   2. No
2. ¿Tu celular es un Smartphone que tiene aplicaciones (WhatsApp, Instagram, Tiktok, etc)?
   1. Si
   2. No
3. Cuéntanos con qué frecuencia realizas las siguientes actividades en su teléfono celular en un día promedio

|  | Nunca | Muy pocas veces al día | Pocas veces al día | Muchas veces al día | Todo el día |
| --- | --- | --- | --- | --- | --- |
| Hacer o recibir llamadas de voz |  |  |  |  |  |
| Escribir o leer mensajes de texto (SMS) |  |  |  |  |  |
| Chatear por WhatsApp |  |  |  |  |  |
| Chatear en otras aplicaciones (Instagram, Snapchat, TikTok, Messenger, etc) |  |  |  |  |  |
| Hacer o recibir video llamadas (por WhatsApp u otras aplicaciones) |  |  |  |  |  |
| Enviar o recibir correos electrónicos |  |  |  |  |  |
| Navegar por internet |  |  |  |  |  |

1. Cuéntanos que tanto te interesaría recibir la siguientes ayudas sobre PrEP a través del teléfono celular:

|  | No estoy interesado | Estoy medianamente interesado | Estoy interesado | Estoy bastante interesado | Estoy muy interesado |
| --- | --- | --- | --- | --- | --- |
| Información general sobre PrEP: qué es PrEP, para qué sirve, cómo se usa |  |  |  |  |  |
| Información más detallada sobre PrEP: Saber si soy un buen candidato para PrEP, qué efectos secundarios tiene, qué necesitaría hacer para tomar PrEP? |  |  |  |  |  |
| Información sobre lugares donde brindan PrEP, horarios, costos y frecuencia de citas |  |  |  |  |  |
| Mensajes recordatorios para las citas de PrEP |  |  |  |  |  |
| Mensajes recordatorios para la toma de pastillas de PrEP |  |  |  |  |  |
| Interactuar con un “asistente virtual” sobre sus dudas sobre el PrEP |  |  |  |  |  |
| Interactuar con una persona de forma virtual sobre sus dudas sobre el PrEP |  |  |  |  |  |
| Que una aplicación me envié notificaciones para hacerme recordar que es la hora de tomar mi pastilla de PrEP |  |  |  |  |  |
| Que una aplicación me permita llevar un registro de mis tomas diarias de PrEP |  |  |  |  |  |
| Usar una aplicación que me permita llevar un registro de mi actividad sexual |  |  |  |  |  |
| Usar una aplicación que me permita saber si estoy protegido del VIH en base a mis regisitros de uso de PrEP |  |  |  |  |  |

1. ¿Con que frecuencia te gustaría recibir información sobre PrEP en su celular?
   1. Semanal
   2. Mensual
   3. Cada 3 meses
   4. Sólo cuando usted nos la pida
   5. Nunca
2. Supongamos que estás planeando empezar PrEP o que ya estás tomando PrEP. ¿Te gustaría poder agendar sus citas para PrEP desde su celular?
   1. Si
   2. No
3. Supongamos que ya tienes agendada una cita para PrEP. ¿Te gustaría recibir recordatorios de tu(s) cita(s) de PrEP en tu celular?
   1. Si
   2. No
4. ¿Con que frecuencia te gustaría recibir recordatorios de tu(s) cita(s) de PrEP en tu celular? (opción múltiple)
   1. Una semana antes de su cita
   2. Dos días antes de su cita
   3. El día previo a la cita
   4. El mismo día de su cita
5. Supongamos que ya estás tomando PrEP. ¿Con que frecuencia te gustaría recibir recordatorios para la toma de tus pastillas de PrEP en tu celular?
   1. Nunca
   2. Diario
   3. Inter-diario
   4. Semanal
6. ¿Qué método preferirías para recibir información o ayudas sobre PrEP en tu celular?
   Ordena las siguientes opciones según tu preferencia (1= Más preferida y 5 = Menos preferida)
   1. Llamadas telefónicas
   2. Mensajes de texto (SMS)
   3. WhatsApp
   4. Chat de otras aplicaciones (Messenger, Snapchat, etc)
   5. Una aplicación creada para ayudarme con el uso de PrEP
7. ¿Estarías de acuerdo con los siguientes canales desde tu celular para comunicarte con el personal del centro donde recibes PrEP?:

|  | Si | No |
| --- | --- | --- |
| Llamadas telefónicas |  |  |
| Mensajes de texto (SMS) |  |  |
| WhatsApp |  |  |
| Chat de otras aplicaciones (Messenger, Snapchat, etc) |  |  |
| Una aplicación creada para ayudarme con el uso de PrEP |  |  |

*Gracias por completar este cuestionario. Tus respuestas nos ayudarán a entender mejor cómo incentivar el uso de PrEP entre las personas que lo necesitan. Si deseas obtener más información sobre PrEP o sobre cómo acceder al PrEP, puedes ponerte en contacto con nosotros:*
