# Supplementary material for: Preferences for mHealth Features to Support Engagement in the HIV Preexposure Prophylaxis Cascade Among Men Who Have Sex With Men in Peru: Cross-Sectional Online Survey
Source: JMIR Public Health Surveill. 2026 Jul 14;12:e84982. doi: 10.2196/84982 (PMC13367761; doi:10.2196/84982)

| **Table S1.** Sociodemographic and behavioral characteristics of HIV-negative men who have sex with men participating in a cross-sectional online survey assessing communication technology use and preferences for mHealth features to support HIV pre-exposure prophylaxis (PrEP) engagement in Peru (June 16–August 31, 2023; N=600), stratified by recruitment method. | | | | | |
| --- | --- | --- | --- | --- | --- |
| **Characteristic** | **Overall**  N = 600, n (%) | **Grindr ads**  n = 79, n (%) | **Instagram and**  **Facebook ads**  n = 216, n (%) | **Organic Social  Media Content^a^**  n = 305, n (%) | **P value^b^** |
| **Age (years)** | 29 (24, 35) | 34 (28, 42) | 30 (24, 36) | 28 (24, 34) | <0.001 |
| **Age (categorical)** |  |  |  |  | 0.129 |
| <25 years | 152 (25.3%) | 13 (16.5%) | 60 (27.8%) | 79 (25.9%) |  |
| ≥25 years | 448 (74.7%) | 66 (83.5%) | 156 (72.2%) | 226 (74.1%) |  |
| **Monthly income** |  |  |  |  | 0.213 |
| <1000 PEN | 197 (32.8%) | 22 (27.8%) | 80 (37.0%) | 95 (31.1%) |  |
| ≥1000 PEN | 403 (67.2%) | 57 (72.2%) | 136 (63.0%) | 210 (68.9%) |  |
| **Educational level** |  |  |  |  | 0.323 |
| Elementary or Secondary | 121 (20.2%) | 12 (15.2%) | 50 (23.1%) | 59 (19.3%) |  |
| Post-secondary | 479 (79.8%) | 67 (84.8%) | 166 (76.9%) | 246 (80.7%) |  |
| **Location** |  |  |  |  | <0.001 |
| Metropolitan Lima | 450 (75.0%) | 48 (60.8%) | 116 (53.7%) | 286 (93.8%) |  |
| All other cities | 150 (25.0%) | 31 (39.2%) | 100 (46.3%) | 19 (6.2%) |  |
| **Owns a smartphone** |  |  |  |  | 0.478 |
| No | 11 (1.8%) | 1 (1.3%) | 6 (2.8%) | 4 (1.3%) |  |
| Yes | 589 (98.2%) | 78 (98.7%) | 210 (97.2%) | 301 (98.7%) |  |
| **Perceived HIV risk** |  |  |  |  | <0.001 |
| No | 160 (26.7%) | 24 (30.4%) | 80 (37.0%) | 56 (18.4%) |  |
| Yes | 440 (73.3%) | 55 (69.6%) | 136 (63.0%) | 249 (81.6%) |  |
| **Engaged in anal sex^c^** |  |  |  |  | <0.001 |
| No | 124 (20.7%) | 8 (10.1%) | 32 (14.8%) | 84 (27.5%) |  |
| Yes | 476 (79.3%) | 71 (89.9%) | 184 (85.2%) | 221 (72.5%) |  |
| **Anal sex partners^c^** |  |  |  |  | 0.001 |
| ≤5 partners | 431 (71.8%) | 50 (63.3%) | 174 (80.6%) | 207 (67.9%) |  |
| >5 partners | 169 (28.2%) | 29 (36.7%) | 42 (19.4%) | 98 (32.1%) |  |
| **Condom use^c^** |  |  |  |  | 0.132 |
| Always | 246 (41.0%) | 35 (44.3%) | 77 (35.6%) | 134 (43.9%) |  |
| Not always | 354 (59.0%) | 44 (55.7%) | 139 (64.4%) | 171 (56.1%) |  |
| **Transactional sex^c^** |  |  |  |  | 0.412 |
| No | 505 (84.2%) | 69 (87.3%) | 185 (85.6%) | 251 (82.3%) |  |
| Yes | 95 (15.8%) | 10 (12.7%) | 31 (14.4%) | 54 (17.7%) |  |
| **Recent STI diagnosis^c^** |  |  |  |  | <0.001 |
| No | 432 (72.0%) | 52 (65.8%) | 176 (81.5%) | 204 (66.9%) |  |
| Yes | 168 (28.0%) | 27 (34.2%) | 40 (18.5%) | 101 (33.1%) |  |
| PEN: Peruvian Soles (1000 PEN: approximately US $268 as of August 15, 2023); STI: bacterial sexually transmitted infection (chlamydia, gonorrhea, or syphilis).  **^a^**Organic content refers to standard posts shared via the participating HIV/AIDS service organizations’ social media accounts and WhatsApp groups for study recruitment, without paid promotion.  **^b^**P values correspond to χ² tests, except where expected cell counts were <5, in which case Fisher’s exact test was used.  **^c^**Behavioral variables refer to the past 6 months. | | | | | |

**Figure S1.** Frequency of use of smartphone-based communication channels among men who have sex with men (cross-sectional online survey, June 16–August 31, 2023; N=600).


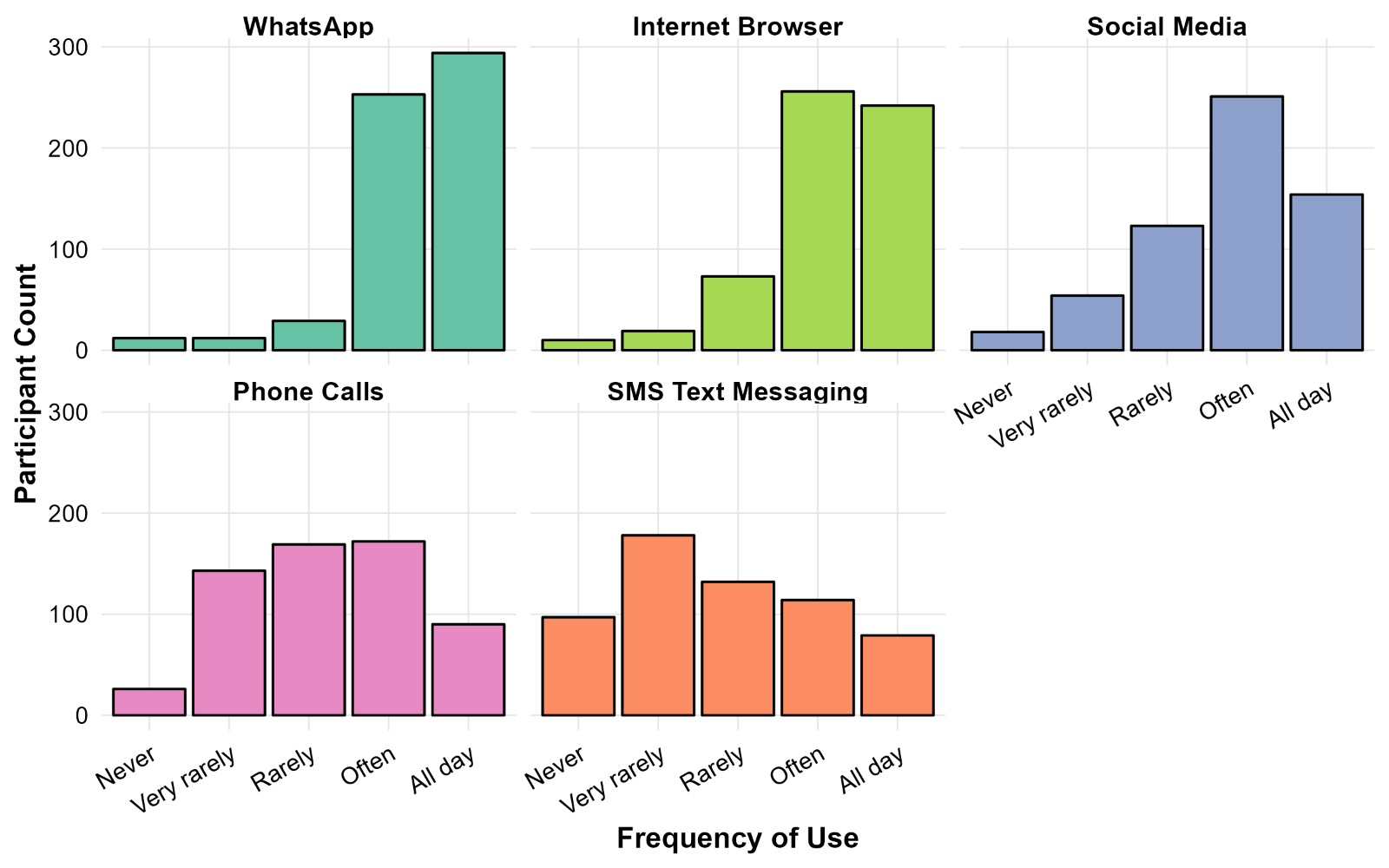


**Figure S2.** Proportion of participants ranking each mHealth communication channel by preference for HIV pre-exposure prophylaxis support among men who have sex with men (cross-sectional online survey, June 16–August 31, 2023; N=600).


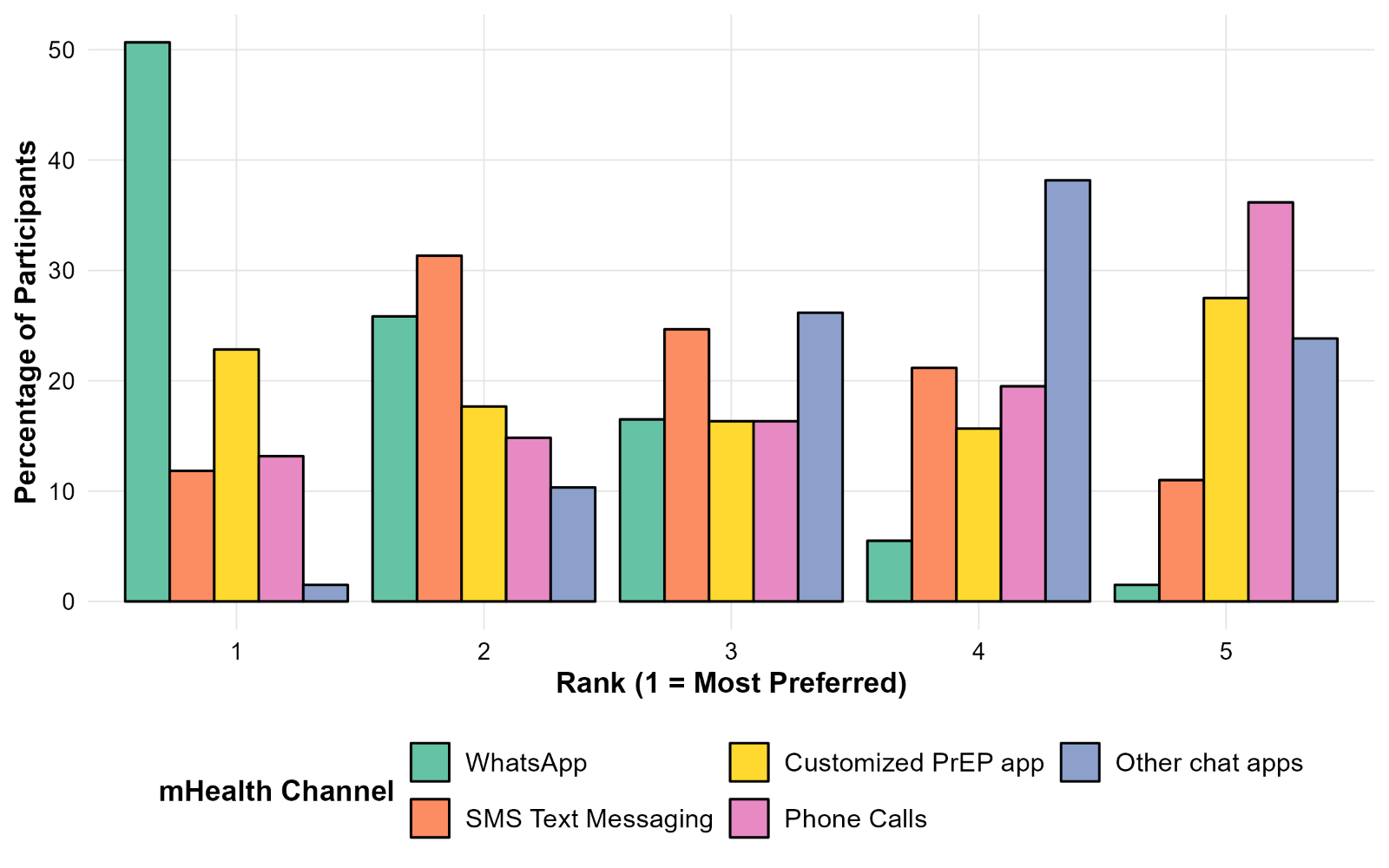


**Figure S3.** Participant interest in specific mHealth features for HIV pre-exposure prophylaxis support by geographical location among men who have sex with men (cross-sectional online survey, June 16–August 31, 2023; N=600).


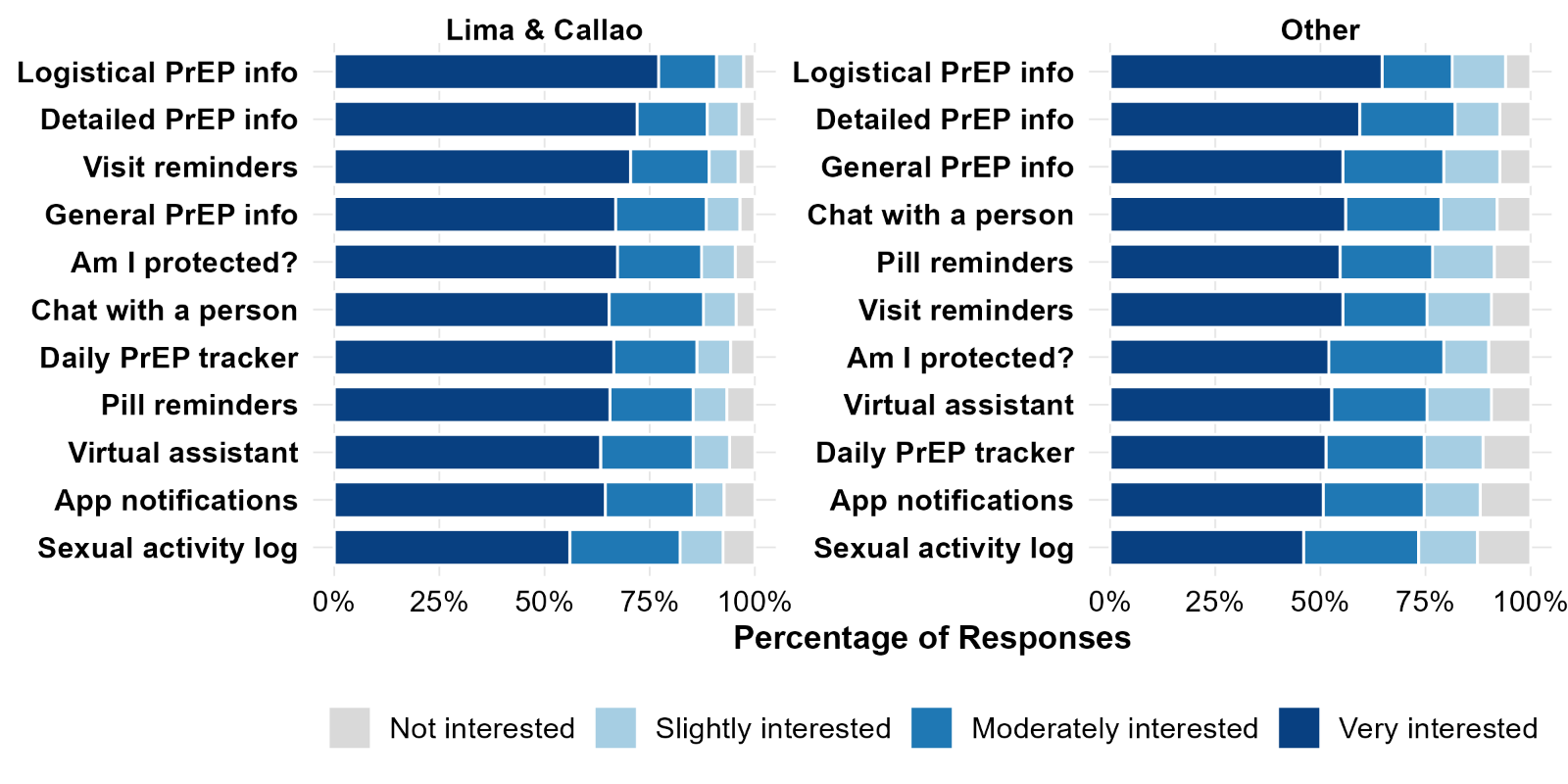


**Figure S4.** Participant interest in specific mHealth features for HIV pre-exposure prophylaxis support by recruitment method among men who have sex with men (cross-sectional online survey, June 16–August 31, 2023; N=600).


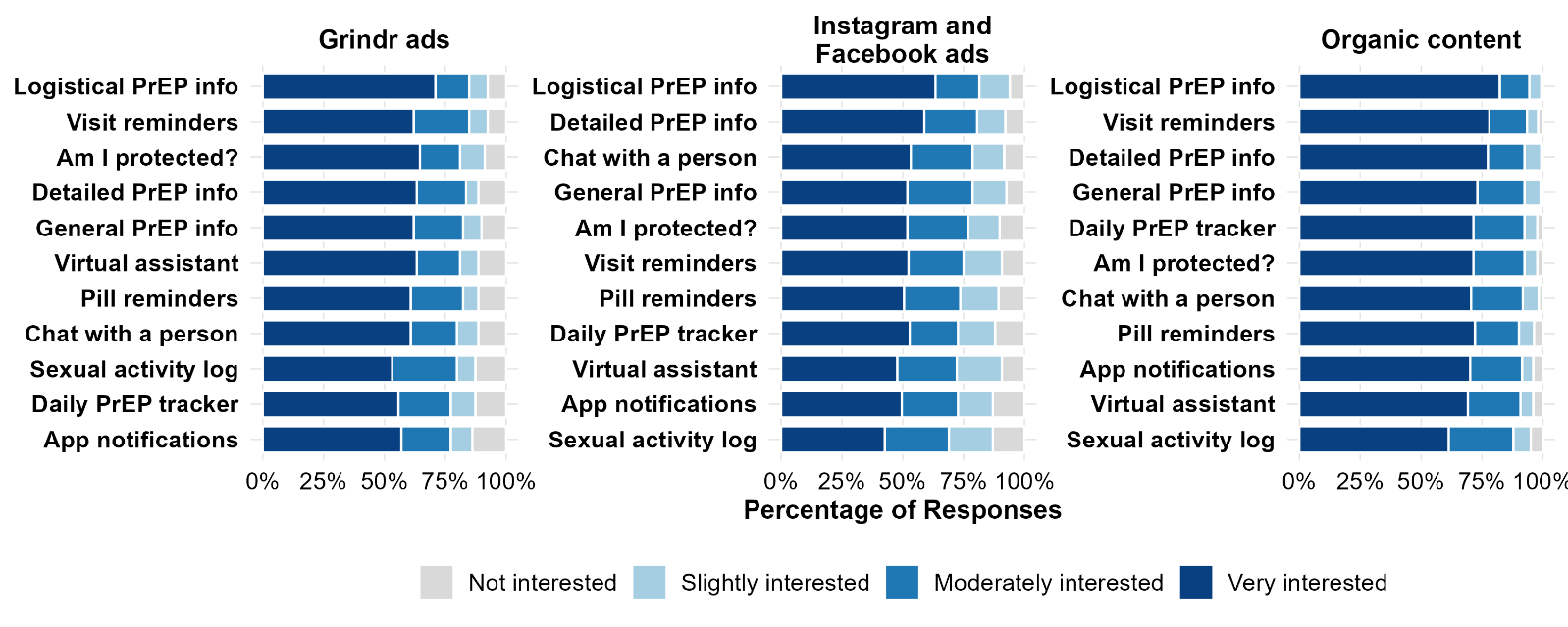

Supplement: Multimedia Appendix 2 [file publichealth-v12-e84982-s002.docx]
